# Postpartum Hypophysitis: A Case Report and a Literature Review

**DOI:** 10.7759/cureus.59396

**Published:** 2024-04-30

**Authors:** Bidisha Baral, Monica Sharma, Ranjan Khadka, Ossama Naveed, Ammer Bekele

**Affiliations:** 1 Medicine, Saint Agnes Hospital, Baltimore, USA; 2 Internal Medicine, Saint Agnes Hospital, Baltimore, USA; 3 Internal Medicine, Ross University School of Medicine, New Jersey, USA

**Keywords:** adrenal insufficiency, postpartum, hypothyroidism, hypopitutarism, auto-immune hypophysitis

## Abstract

Hypophysitis is a rare pituitary gland disease primarily seen in females of reproductive age. Patients can present with various non-specific symptoms, which makes diagnosis challenging. Appropriate endocrine workup supplemented with magnetic resonance imaging (MRI) helps establish a diagnosis. We present a case of a 22-year-old postpartum female who came with nausea, vomiting, and abdominal pain. Global endocrine insufficiency was seen in the laboratory workup, and an MRI confirmed the diagnosis of hypophysitis. She was treated with steroid and thyroid hormone supplementation.

## Introduction

Hypophysitis is a rare cause of hypopituitarism. An annual incidence of hypophysitis is reported to be one in seven to nine million [1]. There are various categories of hypophysitis based on mechanisms, histology, and anatomy. Based on the histology, categories of hypophysitis include lymphocytic, granulomatous, xanthomatous, plasmacytic, necrotizing, and mixed. Lymphocytic hypophysitis (LHy) is the most common type of primary hypophysitis. It accounts for 76-86% of cases of primary subtype [2]. It is autoimmune in origin but is found to be more common during pregnancy and the early postpartum period [2]. Additionally, it is associated with other autoimmune diseases, like Graves disease, Hashimoto Disease, and Addison’s disease [2].

The next most common is granulomatous, which accounts for 20% of the cases, is found to have a female preponderance, and is associated with systemic granulomatous diseases like Wegener granulomatosis and tuberculosis. Additionally, a rare type of primary hypophysitis predominantly affecting the female population is xanthomatous hypophysitis, histologically different from other subtypes (infiltration of foamy histiocytes) but clinically can be similar to LHy [3]. Gadolinium-enhanced magnetic resonance imaging (MRI) is very helpful and helps prevent invasive diagnosis with biopsy [4].

Hypophysitis is a very serious illness and presents with vague symptoms like headaches that demand early recognition to prevent progression to more devastating outcomes. With the varying degrees of hypopituitarism, it is pertinent to recognize the life-threatening complications like adrenal crisis and ways to mitigate that when patients have severe concurrent central hypothyroidism. This case report emphasizes the importance of recognizing the clinical signs and symptoms and the need for more research and diagnostic tools in this area of focus.

## Case presentation

A 22-year-old female without significant past medical history presented with right upper quadrant pain associated with on and off nausea, vomiting, and loss of appetite for about two weeks. She had an uncomplicated vaginal delivery about five months prior and has been amenorrheic since then, despite not breastfeeding, as she had no milk production. She also complained of significant hair loss, fatigue, and weight loss for a couple of months. Her family history was significant for systemic lupus erythematosus in her mother and maternal grandmother. She denied any headache or blurring of vision. While in the Emergency Department, the patient was found to have initial blood glucose of 26 mg/dL, hypokalemia, and episodes of hypotension without any concern for infection. A computerized tomography (CT) scan of the abdomen revealed normal pancreas and adrenal glands. Secondary to the cholestatic pattern of hyperbilirubinemia, magnetic resonance cholangiopancreatography (MRCP) was performed, which ruled out hepatobiliary disease. Endocrine workup was done as depicted in Table 1.

**Table 1:** Laboratory results showing the endocrine workup. ACTH, adrenocorticotropic hormone; TSH, thyroid-stimulating hormone; LH, luteinizing hormone; FSH, follicle stimulating hormone

Tests	Results	Reference value
Random cortisol	<1.0 mcg/dL	5-25 mcg/dL
ACTH level	<1.5pg/mL	7.2-63.3 pg/mL
Cortisol response to ACTH	Baseline, <1.0 mcg/dL; 30 minutes, 2.1 mcg/dL; 60 minutes: 2.9 mcg/dL	
TSH	14.361 mIU/L	0.35-4.94 mIU/L
Total T3	<40 ng/dL	40-193 ng/dL
Free T4	<0.42 ng/dL	0.70-1.48 ng/dL
Aldosterone	<1 ng/dL	0.0-0.30 ng/dL
Serum-connecting peptide (C-peptide)	0.6 ng/mL	1.1-4.4 ng/mL
FSH	5.4 mIU/mL	3.5-12.5 mIU/mL
LH	2.7 mIU/mL	2.4-12.6 mIU/mL

**Figure 1:**
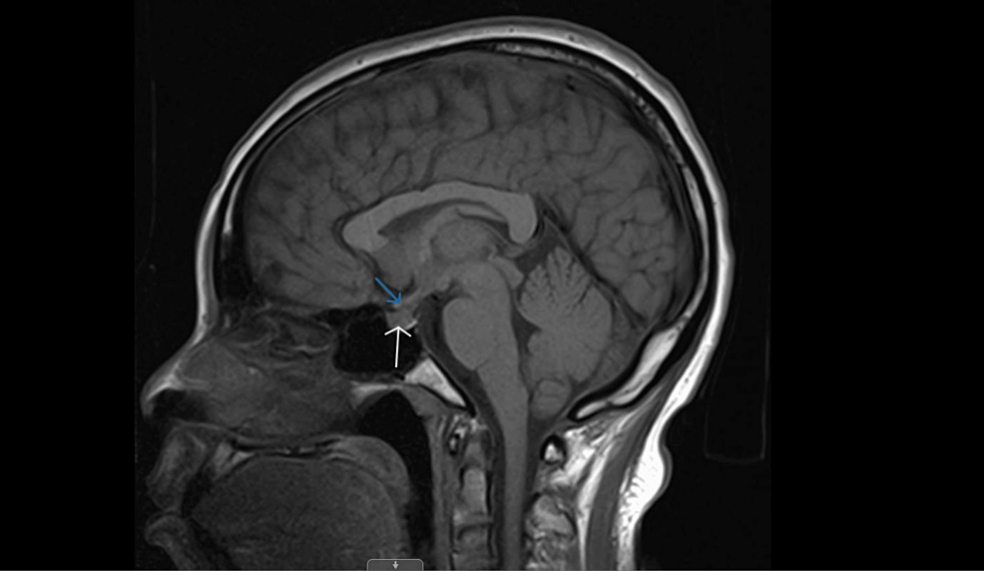
Brain MRI, sagittal section. White arrows: pituitary gland; blue arrows: pituitary stalk. MRI shows a slightly heterogeneous enhancement of the pituitary gland that measured 8 mm and slight thickening of the pituitary stalk inferiorly, features suggestive of hypophysitis. MRI, magnetic resonance imaging

Interpreting the results, adrenal insufficiency (AI) and hypothyroidism were ascertained; the patient was then promptly started on hydrocortisone intravenous (IV) 100 mg stat dose, followed by 50 mg every eight hours and levothyroxine 100 mcg once a day. Low adrenocorticotropic hormone (ACTH) indicated central etiology. Thyroid-stimulating hormone (TSH) was also elevated, with low free thyroxine (T4) and total triiodothyronine (T3), which created ambiguity, as central vs primary hypothyroidism could not be excluded. In the absence of palpable thyroid nodules, thyroid ultrasound was not performed as it would have added to the management. Therefore, an autoimmune workup, including an antinuclear antibody, thyroid peroxidase antibody, and complements 3 and 4, was done, and the results were negative. Based on multiple hormonal deficiencies and the postpartum period, an MRI of the brain (Figure 1) was done to rule out any central etiologies like hypophysitis or Sheehan's syndrome, which revealed a slightly heterogeneous enhancement of the pituitary gland that measured 8 mm and slight thickening of the pituitary stalk inferiorly suggestive of hypophysitis. Fludrocortisone was added along with the continuation of hydrocortisone and thyroid hormone supplementation, with significant improvement in hypotension and fatigue and no further hypoglycemic episodes. The patient was eventually discharged on oral hydrocortisone 20 mg in the morning and 10 mg in the evening, fludrocortisone 0.05 mg once a day, and levothyroxine 100 once a day, with a recommendation to follow-up with an endocrinologist outpatient. 

## Discussion

Hypophysitis is a rare inflammation of the pituitary gland and the infundibulum, posing diagnostic challenges due to its resemblance to other pituitary disorders, such as adenomas and rare metastases [5]. The disorder is categorized into primary, where it stands as an isolated condition including autoimmune types like LHy, and secondary, resulting from systemic diseases, infections, tumors, or medications. Its histological classification includes lymphocytic, granulomatous, xanthomatous, immunoglobulin G4-related disease (IgG4-RD), necrotizing, and mixed forms, with LHy being the predominant type, especially in women [6]. 

The spectrum of hypophysitis symptoms ranges widely from no symptoms to severe manifestations: hypopituitarism, pituitary apoplexy, and potentially fatal adrenal crisis [7]. In patients with primary hypophysitis, a female majority (2/3) was noted. Fatigue (52%), headache (38%), and diabetes insipidus (38%) were noteworthy, and 42% had a concomitant autoimmune disease. The corticotropic, thyrotropic, gonadotropic, and somatotropic axes were impaired in 67%, 57%, 52%, and 20%, respectively. Gonadotropic axes were found to be more frequently affected in men. The frequently encountered sequence of hormonal deficiencies observed with other causes of panhypopituitarism, loss of growth hormone (GH), followed by luteinizing hormone/follicle-stimulating hormone (LH/FSH), TSH, and ACTH, has also been observed with hypophysitis [8-10]. 

The diagnostic approach for hypophysitis is multifaceted, involving biochemical testing for pituitary and peripheral gland hormones, imaging, particularly MRI, which identifies abnormalities in most cases of primary and a significant number of secondary hypophysitis, and the investigation of secondary causes. Pituitary hormones requiring evaluation include serum ACTH, TSH, LH, FSH, GH, and prolactin [11,12]. Typical MRI findings in hypophysitis and their frequencies include pituitary enlargement observed in 23% to 93.2% of cases, uniform contrast enhancement of the pituitary in 23% to 91.7%, pituitary stalk thickening, defined as an anteroposterior diameter greater than 4 mm in the sagittal section in 33.8% to 96% of instances, and the absence of the posterior pituitary bright spot in T1-weighted imaging found in 18% to 70.8% of cases [13-18].

Given the rarity and diverse presentation of hypophysitis and paucity of clinical trials comparing the efficacy of various treatment modalities, there is no consensus on a standardized treatment approach. Treatment strategies involve correcting hormonal deficiencies and alleviating neurological symptoms. Observation is suitable in cohorts with minor symptoms, whereas hormone replacement therapy is essential for addressing deficiencies [13]. In particular, AI is treated with hydrocortisone, and patient education on AI management is provided, including the protocol for emergency hydrocortisone injections. Central hypothyroidism is managed with levothyroxine, with AI treatment prioritized to avoid the risk of adrenal crisis induced by thyroid hormone therapy. Krishnappa et al.'s systematic review and meta-analysis revealed enhanced recovery of visual fields and the corticotropin axis with intravenous glucocorticoids administered at very high doses (over 100 mg/day) and for prolonged periods (longer than 6.5 weeks), especially in severe cases of hypophysitis [19]. The study indicated the potential benefits of high-dose oral glucocorticoids for treating mild to moderate primary adrenal hypophysitis (PAH) [19]. Furthermore, larger prospective studies to evaluate glucocorticoid treatment across different severities and MRI subtypes of the disease, highlighting the need for a consistent hormonal evaluation and comprehensive management strategies of adverse effects, seem paramount. Similarly, in the study by Donegan et al., glucocorticoids led to the highest rate of anterior pituitary hormone recovery (45.5%), outperforming both surgical intervention (14%) and observation alone (21%), albeit with an increased risk of disease progression or relapse in some patients [20]. Immunotherapy with rituximab, azathioprine, mycophenolate, and methotrexate may be a consideration in glucocorticoid-refractory and also as glucocorticoid-sparing modalities. Conventional surgical treatment is now primarily reserved for diagnostic biopsies or cases where there is a need to switch to immunomodulatory treatments due to disease progression or relapse [2].

## Conclusions

In conclusion, our case recognizes the challenges in diagnosing and treating hypophysitis. It is important to have a high index of suspicion in postpartum patients with vague symptoms and multiple endocrine insufficiencies. The current guidelines mainly focus on addressing the hormonal deficiency caused by hypophysitis. Further research is needed to create a diagnostic and treatment approach to improve clinical practice and streamline methods to identify symptomatic patients.
